# Multisystem Inflammatory Syndrome in an Adult (MIS-A) Successfully Treated with Anakinra and Glucocorticoids

**DOI:** 10.3390/microorganisms9071393

**Published:** 2021-06-28

**Authors:** Paolo Cattaneo, Alessandro Volpe, Chiara Simona Cardellino, Niccolò Riccardi, Giulia Bertoli, Tamara Ursini, Arjola Ustalli, Giovanni Lodi, Ivan Daroui, Andrea Angheben

**Affiliations:** 1 Department of Infectious-Tropical Diseases and Microbiology, IRCCS Sacro Cuore Don Calabria Hospital, 37024 Negrar di Valpolicella, Italy; chiara.cardellino@sacrocuore.it (C.S.C.); niccolo.riccardi@yahoo.it (N.R.); giulia.bertoli@sacrocuore.it (G.B.); tamara.ursini@sacrocuore.it (T.U.); andrea.angheben@sacrocuore.it (A.A.); 2 Department of Diagnostic and Public Health, Division of Infectious Diseases, University of Verona, 37129 Verona, Italy; 3 Rheumatology Unit, IRCCS Sacro Cuore Don Calabria Hospital, 37024 Negrar di Valpolicella, Italy; alessandro.volpe@sacrocuore.it; 4 Pneumology Unit, IRCCS Sacro Cuore Don Calabria Hospital, 37024 Negrar di Valpolicella, Italy; arjola.ustalli@sacrocuore.it; 5 Intensive Care Unit, IRCCS Sacro Cuore Don Calabria Hospital, 37024 Negrar di Valpolicella, Italy; giovanni.lodi@sacrocuore.it (G.L.); ivan.daroui@sacrocuore.it (I.D.)

**Keywords:** anakinra, COVID-19, MIS-A, multisystem inflammatory syndrome, SARS-CoV-2

## Abstract

During the current SARS-CoV-2 pandemic, a novel syndrome termed “multisystem inflammatory syndrome in children” (MIS-C) has emerged. MIS-C was linked to COVID-19 and shared some features with Kawasaki disease and Toxic Shock Syndrome, with a common pathogenetic substrate of hyperinflammation and cytokine storm. Lately, MIS was also described in adults (≥21 years of age) and named “MIS-A”. There is no consensus about the treatment of MIS-A; successful use of glucocorticoids and immunoglobulins has been reported in case series, but more solid evidence is lacking. Furthermore, the role of biologic agents with proven benefits against COVID-19, MIS-C, or Kawasaki disease is still unexplored. In this report, we detail the clinical picture and the diagnostic process that led to the diagnosis of MIS-A in a 27-year-old man, focusing on its treatment with anakinra and glucocorticoids, which resulted in full recovery. To our knowledge, this is the first report of the successful use of anakinra for MIS-A, a drug that has already proven useful in the treatment of refractive cases of MIS-C. Anakinra may also play a pivotal role for the treatment of MIS-A.

## 1. Introduction

The following case describes the clinical history, treatment, and outcome of an adult who presented with a sepsis-like picture and features resembling Kawasaki disease, with no evidence of ongoing infection and a history of close contact with a case of Coronavirus disease 2019 (COVID-19) twelve weeks earlier. The case fulfilled the new Brighton Collaboration Case Definition for MIS-A [1] and was treated with a combination therapy of methylprednisolone and anakinra, with excellent outcome.

## 2. Case Presentation

A previously healthy 27-year-old Moroccan man, living in Italy for 11 years, presented himself to our emergency room complaining of a two-day history of profound asthenia and headache, associated mild dyspnea, sore throat, itching, and neck and bilateral leg pain. His medical history was only noteworthy for dust allergy and extrinsic asthma. He reported that he had taken only paracetamol for the relief of symptoms during the previous days, which he had already taken in the past with no side effects.

**Figure 1:**
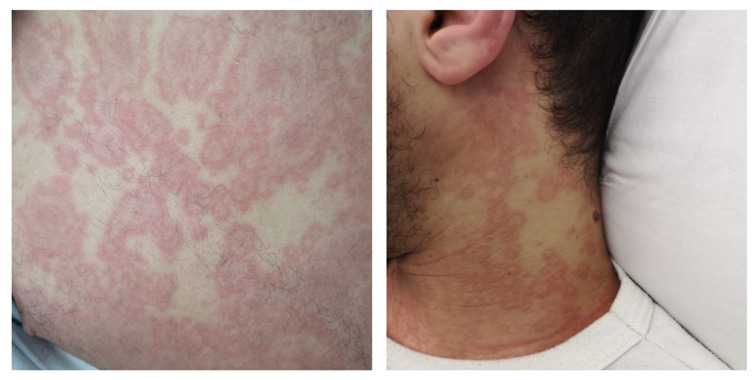
Annular lesions of the trunk and neck.

Clinical examination revealed fever (38.1–38.8 °C) and a widespread maculopapular rash throughout the body; most lesions were annular (Figure 1), and a few lesions resembled furuncles. The patient denied seeing the exanthema previously. Bilateral conjunctivitis, geographic tongue, and nuchal lymphadenopathy were also noticed. Blood pressure, oxygen saturation, and chest X-Ray were all normal. After blood culture collection, the patient was started on empirical antibiotic therapy with ceftriaxone and clindamycin; he was admitted to the Infectious and Tropical Diseases ward for further investigations after a RT-PCR for SARS-CoV-2 on a nasopharyngeal swab proved negative.

Upon admission to our ward, annular lesions similar to erythema multiforme were confirmed (Figure 1), along with moderate lip-edema and enanthem, particularly marked on the dorsal side of the tongue. Progressively, the rash became confluent. Vital signs showed initial hypotension (90/60) and tachycardia (110 bpm), with normal oxygen saturation (98%) and mild tachypnea (21 breaths/min). The patient looked exhausted and showed episodes of vomiting, but he did not have cognitive impairment. He denied recent travel, trauma, insect or animal bites, unprotected sexual intercourses, consumption of homemade food, history of drug or alcohol abuse, medicines intake, or drug allergies. He lived with his parents and revealed that his mother had been diagnosed with COVID-19 twelve weeks earlier, while he had tested negative for SARS-CoV-2 at the swab performed at that time.

A few hours after admission, the clinical conditions rapidly deteriorated, evolving into shock and oliguria refractive to fluid resuscitation; the patient was transferred to the intensive care unit and the antibiotic therapy was empirically switched to cefepime plus doxycycline. Hydrocortisone 1 g IV was administered due to hypotension.

Blood exams drawn at presentation in the emergency room showed leukocytosis with left shift (10,700 WBC/mm^3^; neutrophils, 9800/mm^3^; and lymphocytes, 500/mm^3^) and mild thrombocytopenia (123,000 platelets/mm^3^), along with elevation of C-reactive protein (151 mg/L), procalcitonin (1.29 ng/mL), and D-Dimer (1198 ng/mL). Liver and kidney function was normal.

After the development of severe hypotension, inflammation biomarkers skyrocketed, with C-reactive protein reaching 278 mg/L; procalcitonin, 15.86 ng/mL; and D-Dimer, 14,556 ng/mL. The blood count was notable only for further reduction in platelet count (106,000/mm^3^), while creatinine worsened to 115 µmol/L (from 79 µmol/L). INR was 1.7, fibrinogen was not consumed (5.88 g/L), and ferritin was only mildly elevated (362 µg/L; upper normal value, 336.2 µg/L); lactate levels were 2.4 mmol/L.

At day 2, new blood samples showed a pronounced elevation in high sensitivity-Troponin I (618 ng/L, normal range <20 ng/L), elevation of BNP (168 pg/mL), and worsening neutrophilia and lymphopenia (22,100 neutrophils/mm^3^; lymphocytes, 200/mm^3^). The exanthema turned erythrodermic and norepinephrine was begun to maintain adequate blood pressure values. Plasma transfusions were also performed in an effort to correct the dyscoagulation. A lumbar puncture was not done due to the absence of meningeal signs or cognitive impairment and concern about the hemorrhagic risk. A whole-body CT scan was performed looking for possible infective foci, but the report was entirely normal, except for bilateral pleural effusion and alveolar filling with thickening of the interlobular septa, consistent with congestive phenomena. An echocardiography excluded signs of endocarditis or lesions on the coronary artery tree.

At day 3, respiratory function worsened due to severe bronchospasm and congestion; the patient was supported with non-invasive ventilation and then tracheal intubation. C-reactive protein and procalcitonin were still highly elevated and neutrophilia further increased at 48–60 h from the beginning of broad-spectrum antibiotics; blood cultures and other microbiological tests were negative at this point, hence multisystem inflammatory syndrome with a Kawasaki-like picture was suspected, and IV corticosteroids therapy was started (methylprednisolone, 1 g IV q24h for 3 days). At day 4, anakinra was added and administered for 5 days (100 mg q12h for the first 3 days, then 100 mg q24h for 2 days).

**Figure 2:**
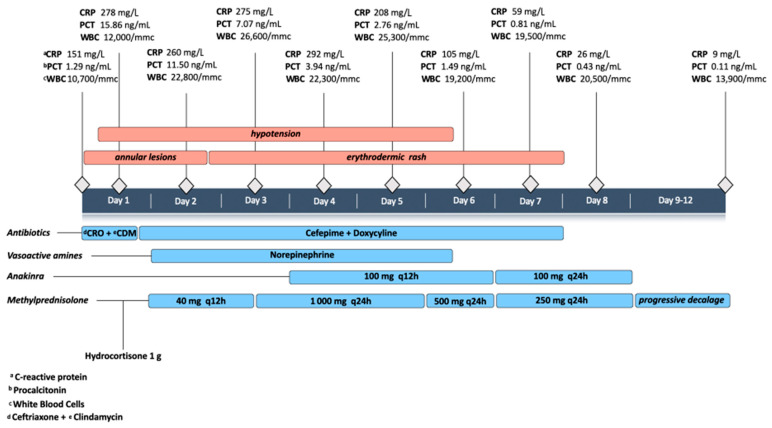
Case timeline.

From day 4, the patient became afebrile, and the clinical conditions improved until discharge from the ICU at day 8; he finally recovered with a complete *restitutio ad integrum* and blood test normalization after 12 days (Figure 2).

All the following tests proved negative: cold agglutinins, multiple blood cultures, urine culture, pharyngeal swab for streptococcus, bronchoalveolar lavage culture, rectal swab for multi-drug resistant bacteria, RT-PCR for SARS-CoV-2 on nasopharyngeal swab and on bronchoalveolar lavage, PCR for enterovirus-RNA on blood, pneumococcal and legionella urinary antigens, Beta-D-Glucan on blood, serologies for SARS-CoV-2, HIV, HCV, HBV, syphilis, borreliosis, *Mycoplasma pneumoniae*, *Coxiella burnetii*, and leptospirosis. Serologies for the following pathogens were consistent with past infection: cytomegalovirus, Epstein–Barr virus, adenovirus, *Chlamydophila pneumoniae*. A nasal swab was positive for methicillin-sensitive *Staphylococcus aureus* colonization. Tryptase and triglycerides were normal. A coronary-computed tomography scan was done and resulted normal.

## 3. Discussion and Conclusions

Since summer 2020, the existence of a multisystem inflammatory syndrome in people aged ≥21 years resembling the one seen in children and adolescents has been increasingly recognized [2,3,4,5,6,7]. The two syndromes share a common, though still largely unknown, pathogenetic pathway based on a state of hyperinflammation and systemic cytokine storm, which leads to rapid clinical deterioration and hemodynamic instability, often accompanied by mucocutaneous manifestations [1,8]. Case reports have described similarities between both MIS-C and MIS-A and Kawasaki Disease (KD) and its severe manifestation of Kawasaki Disease Shock Syndrome (KDSS) [3,4,7,9]. Some divergences have also been underpinned between MIS-C and MIS-A, for example the severity of cardiac dysfunction and the higher incidence of thrombosis in many cases of the latter [2].

Despite several cases reported in the literature, MIS-A lacks a widespread and accepted definition of the syndrome; so far, the case definition made by the U.S. Center for Disease Control (CDC) requires 5 criteria to be present [2]: (1) a severe illness requiring hospitalization in a person aged ≥21 years; (2) a positive test result for current or previous SARS-CoV-2 infection in the last 12 weeks; (3) severe dysfunction of one or more extrapulmonary organ systems; (4) laboratory evidence of severe inflammation; (5) absence of severe respiratory illness (to exclude patients in which inflammation and organ dysfunction might be attributable simply to tissue hypoxia); alternative diagnoses must be excluded. A Brighton Collaboration Case Definition has recently been developed by a group of experts with similar criteria [1], adding the history of close contact with known COVID-19 case within 12 weeks.

The case we have described meets all the previous criteria; indeed, respiratory insufficiency developed late in the course of the disease because of bronchospasm and congestive phenomena and was not present when systemic hyperinflammation and shock had already developed.

Our patient has a SARS-CoV-2 negative serology; however, the antibodies against the virus could have become undetectable in three months [10,11,12] and it is not impossible that only the above-mentioned epidemiological criterion is recorded in MIS-A cases [1].

Our patient proved to be negative for septic shock and a vast number of possible infectious etiologies, as well as for systemic mastocytosis. Hemophagocytic lymphohistiocytosis was also excluded due to the absence of significant bicytopenia, ferritin only mildly elevated, normal triglycerides values, and the absence of splenomegaly. Furthermore, he fulfilled all the criteria for KD, except for the adult age and the fever being observed for less than 5 days.

We must recognize that the possibility that an infectious cause other than SARS-CoV-2, not detected by our tests, might have triggered a systemic dysregulated response of the immune system cannot be entirely ruled out.

The case we reported highlights the importance of considering MIS diagnosis, even among adults, as physicians treating adults might be unaware of KD features or have difficulty contextualizing an apparent septic shock into the correct frame, thus delaying the beginning of an appropriate treatment. In our case, many features were common to sepsis, such as the distributive nature of the shock and the blood count alterations (neutrophilia and lymphopenia with thrombocytopenia). Myocarditis, which has been described in numerous cases of MIS-A [7], was not evident echocardiographically in our patient, although a marked alteration in troponin developed.

To our knowledge, this is the first report describing the use of a blocker of IL-1 receptor, anakinra, in MIS-A, with a very good outcome. Anakinra has been largely used and studied in the treatment of COVID-19 and is part of several clinical trials for this disease [13,14,15]; furthermore, its use has been described in a series of MIS-C cases, with good results [16]. Interestingly, successful use of anakinra in two cases of MIS-C refractive to IVIG and systemic glucocorticoids has also recently been described [17]. It could therefore prove useful for the treatment of MIS-A, a clinical entity for which established clinical practices are still foggy.

## Data Availability

All data are stored in hospital files and electronic medical records and are available within the manuscript; further details, if available, can be obtained from the corresponding author upon request.
